# Genome-Wide Requirements for Resistance to Functionally Distinct DNA-Damaging Agents

**DOI:** 10.1371/journal.pgen.0010024

**Published:** 2005-08-19

**Authors:** William Lee, Robert P St.Onge, Michael Proctor, Patrick Flaherty, Michael I Jordan, Adam P Arkin, Ronald W Davis, Corey Nislow, Guri Giaever

**Affiliations:** 1 Department of Genetics, Stanford University School of Medicine, Stanford, California, United States of America; 2 Department of Biochemistry, Stanford University School of Medicine, Stanford Genome Technology Center, Palo Alto, California, United States of America; 3 Department of Electrical Engineering and Computer Science, University of California, Berkeley, California, United States of America; 4 Physical Biosciences Division, Lawrence Berkeley National Laboratory, Berkeley, California, United States of America; 5 Division of Computer Science, Department of Statistics, University of California, Berkeley, California, United States of America; 6 Howard Hughes Medical Institute, Department of Bioengineering, University of California, Berkeley, California, United States of America; Yale University, United States of America

## Abstract

The mechanistic and therapeutic differences in the cellular response to DNA-damaging compounds are not completely understood, despite intense study. To expand our knowledge of DNA damage, we assayed the effects of 12 closely related DNA-damaging agents on the complete pool of ~4,700 barcoded homozygous deletion strains of *Saccharomyces cerevisiae*. In our protocol, deletion strains are pooled together and grown competitively in the presence of compound. Relative strain sensitivity is determined by hybridization of PCR-amplified barcodes to an oligonucleotide array carrying the barcode complements. These screens identified genes in well-characterized DNA-damage-response pathways as well as genes whose role in the DNA-damage response had not been previously established. High-throughput individual growth analysis was used to independently confirm microarray results. Each compound produced a unique genome-wide profile. Analysis of these data allowed us to determine the relative importance of DNA-repair modules for resistance to each of the 12 profiled compounds. Clustering the data for 12 distinct compounds uncovered both known and novel functional interactions that comprise the DNA-damage response and allowed us to define the genetic determinants required for repair of interstrand cross-links. Further genetic analysis allowed determination of epistasis for one of these functional groups.

## Synopsis

Cells have evolved sophisticated ways to respond to DNA damage. This is critical because unrepaired damage can kill cells or cause them to become cancerous. The response to DNA damage has been studied for more than 50 years, and has been found to be extremely complex. The traditional way of understanding this complexity is to divide the process into its component parts with the goal of eventually reconstituting the entire process. In this study, the authors extend classical approaches using genomics—an approach that involves studying all genes in an organism simultaneously. The authors tested 12 distinct compounds (many used in cancer chemotherapy) that damage DNA and uncovered new genes involved in DNA repair. They then grouped the compounds to define how they attack cells. Using this approach, the study found that many similar DNA-damaging agents act in comparable ways to damage DNA, but surprisingly, similar compounds can also act on cells by very different mechanisms. Specifically grouping the findings together and verifying the significant results lends a high degree of confidence in the data. The development of such a reproducible experimental design is important for inspiring future experiments.

## Introduction

Eukaryotic cells possess multiple mechanisms to cope with structural damage to their DNA. For example, nucleotide excision repair (NER) excises oligonucleotides containing a covalently modified base and resynthesizes the deleted fragment using the undamaged DNA strand as template [1]. Cell-cycle checkpoints respond to damaged DNA by initiating a series of phosphorylation and dephosphorylation events that result in a transient arrest of the cell cycle, providing time for lesions to be repaired [2,3]. During DNA replication, multiple pathways ensure the stability and restarting of stalled replication forks at sites of DNA damage [4,5]. The sum total of these activities and similar functional repair “modules” are commonly referred to as the DNA-damage response (DDR).

The study of gene products and pathways that comprise the DDR has particular relevance to both the etiology and treatment of cancer in man. A causative role for the DDR in carcinogenesis is supported by several observations, including the high degree of genomic instability observed in tumor cells [6,7] and the number of DDR genes that, when mutated, lead to cancer or to one of several inherited disorders characterized by cancer predisposition [8]. Therapeutically, many cytotoxic agents used to treat cancer act by directly targeting DNA. Therefore, pathways that actively repair DNA lesions are likely to contribute to the significant problem of clinical drug resistance [9].

The high degree of conservation between the genes and pathways involved in maintaining genetic integrity in yeast and all metazoans supports the use of model organisms to better understand these pathways in man. Indeed, classical forward genetic screens in *Saccharomyces cerevisiae* have led to the identification of several genes important for responding to DNA damage, providing the parts list from which the current framework of the DDR has emerged [10,11]. The results of these screens have been augmented by the recent disruption and barcoding of each predicted yeast open reading frame which has proven to be a powerful tool in identifying additional components of the DDR [12–18].

In this study, we used fitness profiling [19–22] to interrogate a pooled collection of ~4,700 *S. cerevisiae* homozygous deletion strains for sensitivity to 12 agents known to compromise the structural integrity of DNA (Table 1). Fitness analysis of individual deletion strains identified in these global experiments confirmed our microarray data and revealed that the genetic requirements for resistance to DNA-damaging agents may exceed previous estimates. We discovered that those strains sensitive to these compounds carried deletions primarily in genes known to be involved in DNA metabolism, but we also uncovered genes not previously known to be related to the DDR. While resistance to a given compound typically required multiple DDR modules, we found that the relative importance of these modules was varied, even when comparing functionally related compounds. The significance of our results are 4-fold: (1) we developed a robust exportable assay to identify and confirm DDR components; (2) filtering and clustering the data allowed classification of both the mechanism of drug action and gene function; (3) we used epistasis analysis to identify novel functional relationships between DDR components; and (4) we were able to clearly discriminate the genome-wide response to agents that damage DNA by forming interstrand cross-links (ICLs) from those that do not.

**Table 1:**
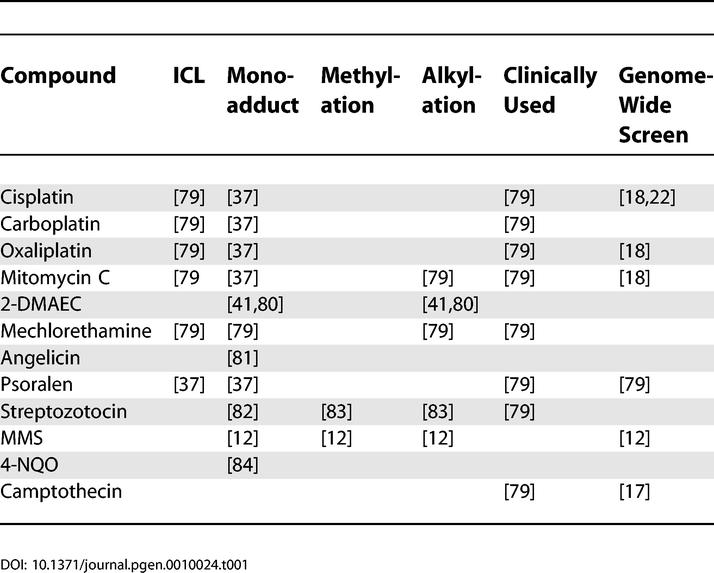
Summary of Compounds, with References Indicated

## Results

### Fitness Profiling of the Yeast Deletion Collection

The yeast deletion collection is a powerful tool for identifying genes important for fitness on a genome-wide scale under a diverse set of environmental conditions [13,16,20,22–24]. This resource has been particularly valuable in the study of cellular mechanisms that respond to DNA-damaging agents [12–14,16,18,25–27]. Each of these studies has provided new insights into the DDR. The underlying protocols in these well-executed studies are, however, so disparate that they preclude any direct comparisons beyond general conclusions. For example, some studies were performed on solid media, while others used high doses of compound followed by recovery in liquid media. Furthermore, the data analysis varies from study to study. To provide a consistent and comprehensive dataset of the DDR, we (1) profiled 12 unique DNA-damaging compounds (six of which had not previously been profiled) (Table 1) using a validated protocol [22]; (2) confirmed a subset of our microarray fitness data by individual strain analysis; and (3) where possible, correlated our results with previously published studies. Specifically, we sought to detect mechanistic differences between compounds that form ICLs (cisplatin, oxaliplatin, carboplatin, mechlorethamine, mitomycin C, and psoralen) and those that do not (angelicin, 4-nitroquinoline-1-oxide [4-NQO], 2-dimethylaminoethyl chloride [2-DMAEC], methyl methanesulfonate [MMS], streptozotocin, and camptothecin).

In our experiments, ~4,700 homozygous diploid deletion mutants were grown in pooled cultures in the presence of compound. Cells were then collected, genomic DNA purified, and the unique molecular barcodes present in each strain amplified by PCR and hybridized to an oligonucleotide array carrying the barcode complements. The relative fitness of each strain was then determined by comparing the signal intensity for each strain on the microarray to the corresponding intensities obtained from a series of no-drug control arrays (see Materials and Methods; Dataset S1; Protocol S1).

### Validating the Approach by Individual Strain Confirmation

**Figure 1:**
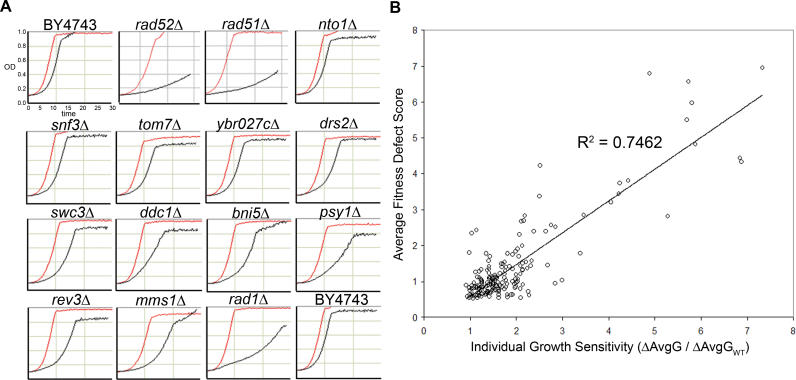
Comparison of Individual Strain Analysis to Microarray Results (A) Representative growth curves of 16 individual strains grown in the presence of solvent alone (DMSO, red curve) and 62.5 μM mechlorethamine (black curve). Growth was monitored by measuring the optical density (OD_600_) of cultures every 15 min for 30 h. The fitness of each strain was defined by the difference between the average doubling times in mechlorethamine and in DMSO (see Materials and Methods). (B) Correlation between growth rates of individual strains and microarray-based fitness estimates. The ratio of growth rates of the 186 individual homozygous deletion strains (the top 233 ranked mechlorethamine-sensitive strains as determined by three replicate microarray experiments minus 47 strains which exhibited a slow-growth phenotype when individually cultured in the absence of mechlorethamine) over an average wild-type growth rate are plotted on the x-axis against the average fitness-defect scores from three pool experiments on the y-axis. The correlation (*R* ^2^ = 0.7462) is highly significant (*p* = 5.4e*−*57).

Little experimental evidence directly addresses how well fitness defects or sensitivities measured by microarray analysis correlate with actual growth rates of individually cultured strains. To directly address this issue, we cultured the 233 deletion strains most sensitive to mechlorethamine individually (as determined from three replicate microarray experiments, see Materials and Methods). The individual growth rates of these strains were measured, both in the presence and absence of mechlorethamine, by taking optical-density readings of liquid cultures every 15 min for 30 h (Dataset S2; Protocol S2). Figure 1A shows representative growth curves for 32 of these cultures (16 in dimethyl sulfoxide [DMSO, diluent control] and 16 in mechlorethamine). We defined the sensitivity to mechlorethamine of each strain by calculating the difference between the average doubling time (AvgG) in DMSO and in mechlorethamine (see Materials and Methods). These values were then normalized to wild-type and plotted against their corresponding fitness-defect scores as measured from the microarray (Figure 1B). We observed a highly significant correlation (*R* ^2^ = 0.5086, *p* = 8.5e−38; data not shown). When we removed strains exhibiting fitness defects in the absence of drug from the analysis, this correlation increased (*R* ^2^ = 0.7462; *p* = 5.4e−57). This is consistent with slow-growing strains yielding artificially low fitness-defect scores in microarray-based fitness analysis of pooled cultures (see Overall Experimental Design). Of 233 individual strains analyzed, 206 exhibited significant mechlorethamine-dependent fitness defects compared to that of a wild-type strain (see Materials and Methods).

To further test how well our significance calling in our microarray experiments correlated with individual growth rates, we compared a calculated false-discovery rate (FDR) [28,29] with a measured FDR for the top 81 most sensitive strains according to the combined results for pooled growth in mechlorethamine (Table 2). The FDR calculation allows us to establish a fitness-defect score threshold for a given acceptable rate of false discovery. Specifically, we calculated the cut-off for several FDRs and examined the individual growth curves of strains that were designated as significant at these cut-offs. For example, the array results show that a 5% FDR cut-off results in 37 sensitive strains from the pooled experiments. The individual growth curves of these 37 strains reveal that 35 of them are indeed sensitive to mechlorethamine, giving us a 5.4% FDR (two out of 37). For this drug, our measured FDR is lower than the calculated FDR. It should be noted that although the above underscores the low FDR in our experiments, it can not address the extent of the false-negative rate.

**Table 2:**
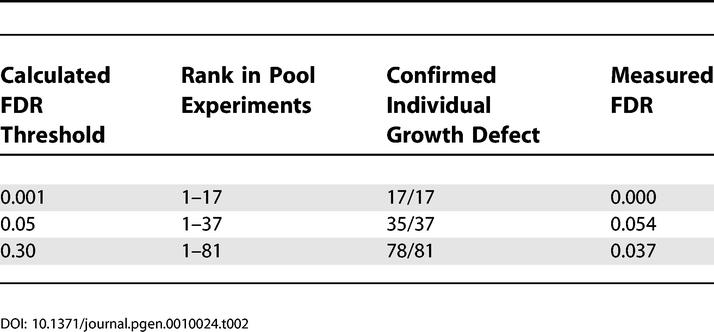
Calculated FDR Versus Experimentally Measured FDR

### Overall Experimental Design

As we refined our assay for the DNA-damaging agents, it became apparent that the length of time for which cells are exposed to compound has a dramatic effect on the sensitivity of deletion strains lacking those genes most required for growth. Specifically, in our standard assay design, frozen aliquots of the deletion collection are recovered in rich media for ten generations until they reach logarithmic growth phase [22]. Compound is then added and the culture is robotically grown for precisely 20 generations. This prolonged chronic exposure allows for detection of those gene products that are required for resistance even for those gene products that have small (<5%) growth defects. We used this standard assay to profile four compounds: mechlorethamine, cisplatin, streptozotocin, and camptothecin. The results of these experiments made it clear that any deletion strain exhibiting a slow-growth phenotype in the absence of compound becomes depleted from the pool even before the compound is applied. As a consequence, this experimental design precludes any meaningful measure of the fitness of this subset of strains. To circumvent this problem, we altered our experimental protocol in the following way: frozen pool aliquots were thawed and immediately exposed to compound for the equivalent of five generations of wild-type growth.

Control experiments showed no non-specific sensitivity of the wild-type strain as a result of cells recovering from a frozen state using this protocol (data not shown). This revised experimental design was used to screen the 12 compounds (see Table 1) because (1) this low number of generations minimizes the false-negative rate resulting from slow-growing strains; and (2) these experiments should identify gene products immediately required for resistance to compound. The advantages of this design are demonstrated by examining the *rad51Δ* and *rad52Δ* homozygous deletion strains, both of which are defective for homologous recombination-mediated repair [30]. Both are acutely sensitive to mechlorethamine but also exhibit a slow-growth phenotype in the absence of mechlorethamine (see Figure 1A). In the standard 20-generation assay, however, the fitness-defect scores of these deletion strains in mechlorethamine ranked 1,676 and 2,051, respectively, among the other ~4,700 strains in the pool (where the strain most sensitive to mechlorethamine has a rank of 1). When the revised experimental design was used, the average rank of these strains increased to 21 and 23, respectively. Therefore, in the former case, neither strain was sensitive, but in the latter case, both were extremely sensitive.

**Figure 2:**
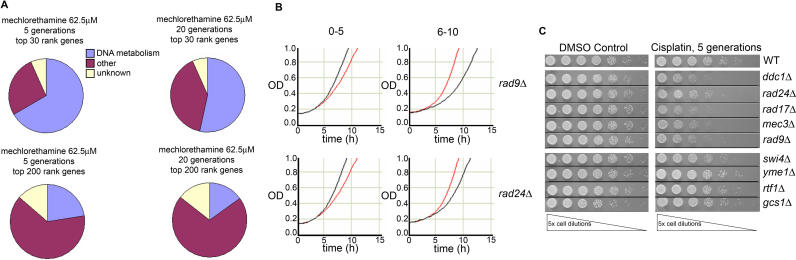
The Effect of Exposure Duration on the Genetic Requirements for Resistance to DNA-Damaging Agents (A) Pie charts showing relative percentage of sensitive genes categorized into either “DNA metabolism”, “unknown”, or “other”. All of the Gene Ontology [63] slim annotations (ftp://ftp.yeastgenome.org/yeast/data_download/literature_curation/go_slim_mapping.tab. Accessed February 17, 2005) are combined into “other” except for those classified in the unknown or DNA-metabolism category. The relative distributions of the mechlorethamine experiments are shown as a function of both time and gene rank. (B) BY4743 (wild-type) and the DNA-damage-checkpoint mutants, *rad9Δ* and *rad24Δ,* were grown in the presence of 500 μM cisplatin over the course of ten population doublings. Yeast cultures were maintained in an exponential phase of growth by robotic dilution of cultures after five doublings into fresh media containing cisplatin. The growth of each deletion strain (black curve) is compared to that of BY4743 (red curve) between 0–5 (left) and 6–10 (right) population doublings. The *rad9Δ* and *rad24Δ* deletion strains exhibit accelerated grow rates in the first five generations, but by ten generations begin to demonstrate slow growth as compared to wild-type. (C) Viability assay of strains treated with cisplatin or DMSO for five generations. Indicated strains were removed from cultures after they reached an OD_600_ of 2.0, and were diluted as shown. Strains were chosen based on the criteria that they did not show a growth defect at five generations but did show a growth defect at 20 generations. Dilutions were pinned onto YPD plates in a 5-fold concentration series. The wild-type parental diploid strain is compared to several diploid deletion strains that exhibited a decrease in viability: *ddc1Δ, rad24Δ, rad17Δ, mec3Δ,* and *rad9Δ.* In contrast, several of these strains showed little or no decrease in viability at five generations of growth: *swi4Δ, yme1Δ, rtf1Δ,* and *gcs1Δ*. This figure underscores the point that, despite the lack of an apparent growth defect in liquid culture, several deletion strains lose viability relatively rapidly when exposed to cisplatin.

Despite our observation that the shorter-generation assay detected many DDR genes, comparing the five-generation assay with the standard 20-generation assay yielded additional insight. For example, there is an increase in strains that are deleted for genes classified as “other” or “unknown” that exhibit fitness defects only after extended exposure (i.e. 20 generations) to compound (Figure 2A). Particularly interesting examples are strains defective in DNA-damage checkpoints. Table 3 summarizes the average rank of five checkpoint-defective strains *(rad9Δ, rad24Δ, rad17Δ, ddc1Δ,* and *mec3Δ)* following five or 20 generations of pool growth in 500 μM cisplatin. We followed up on this result by monitoring the growth rate of these individual strains during the first five and second five generations of growth in cisplatin (Figure 2B). Strikingly, both the *rad9Δ* and *rad24Δ* strains exhibited accelerated growth relative to wild-type during the first five generations, consistent with a lack of checkpoint-mediated cell-cycle arrest [31,32]. Despite this accelerated growth, these strains exhibited reduced viability even at the five-generation point (Figure 2C). Consistent with this observation during the next five generations (6–10), these mutants did exhibit a reduced rate of growth, presumably due to the accumulation of DNA and chromosomal damage associated with uncontrolled progression through the cell cycle. Notably, the growth rate of deletion strains *rad17Δ, ddc1Δ,* and *mec3Δ,* whose protein products form a complex loaded onto DNA at sites of damage in a *RAD24*-dependent manner [33], were indistinguishable from that of a *rad24Δ* strain (Figure 2C; data not shown). These results may explain the discrepancy between conflicting reports that address the requirement of checkpoint genes for resistance to cisplatin [18,34,35].

**Table 3:**
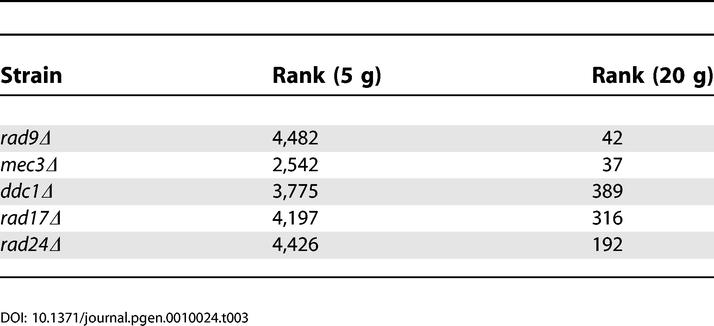
Comparison of the Average Ranked Sensitivity of Five DNA-Damage-Checkpoint Mutants from Experiments in which the Pooled Homozygous Deletion Collection Was Exposed to 500 μM Cisplatin for Either Five or 20 Generations

In addition to checkpoint-defective strains, a number of other deletion strains exhibited sensitivity only after 20 generations of growth. These strains, however, do not exhibit accelerated growth at five generations but appear to be required only after long-term chronic exposure. These strains are deleted for genes that modify chromatin structure and are involved in the respiratory chain (Table S1). Despite the lack of an observable growth defect at five generations, we hypothesized that some of these strains, depending on the cause of their sensitivity, may have reduced viability. To test this, we plated serial dilutions of individual strains (that exhibited sensitivity after 20 generations, but not after five generations) on yeast extract/peptone/dextrose (YPD) following five generations of growth in 500 μM cisplatin (Figure 2C). Of the four we tested, *gcs1Δ, swi4Δ, rtf1Δ,* and *yme1Δ,* none showed an increased loss of viability. Liquid cultures of these strains confirmed these results, as a decrease in growth rate compared to wild-type is only observed at later-generation time points, and is not observed at the five-generation time point.

### Interstrand DNA Cross-Linkers

In this study, we examined the effects of a variety of DNA-damaging agents, but placed particular emphasis on compounds that induce cross-links between complementary strands of DNA (the ICLs). Even though each of the six ICL-inducing compounds we profiled cause a variety of structural damage to DNA (see Table 1), the cytotoxic effects of these compounds are attributed primarily to ICLs [36]. These compounds differ in the efficacy with which they induce ICLs, ranging from 30–40% of all lesions for psoralen to 1–5% of all lesions for mechlorethamine [37]. They also differ in their preferred substrates. Mitomycin C, for example, typically cross-links guanines at CpG sequences [38], whereas psoralen predominantly cross-links thymines at TpA sequences [39].

**Figure 3:**
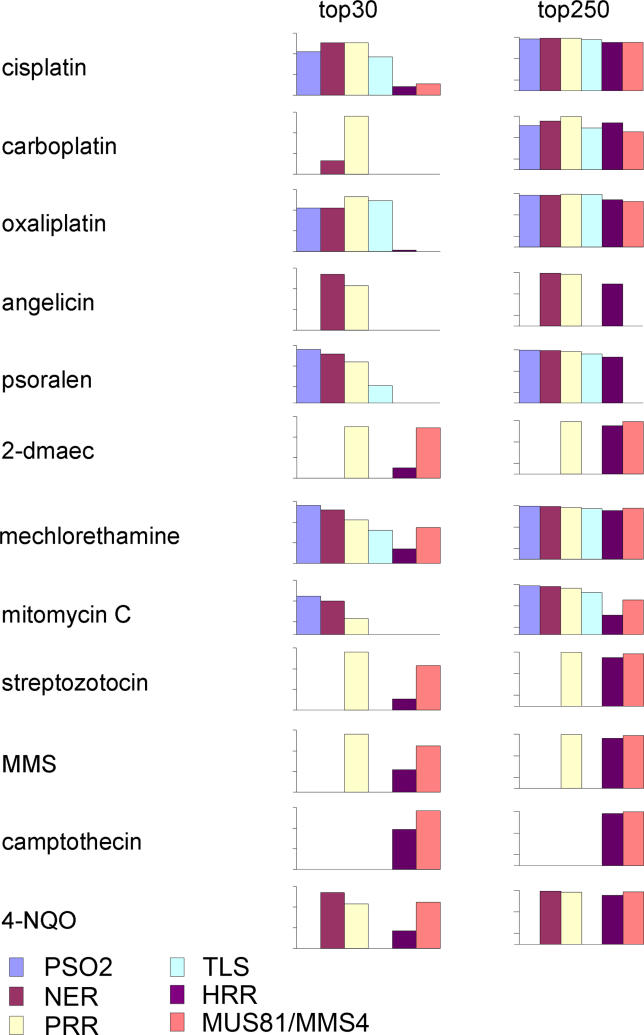
The Relative Importance of Well-Characterized DDR Modules for Resistance to Different DNA-Damaging Agents Detailed examination of strains with mutations in DNA-damage-response genes. Each bar graph represents only strains that were found to be among the top 30 (or 250) most sensitive strains in that compound and are known to be members of a well-characterized DNA-damage-response pathway. The bars represent the median rank for genes in each of the gene groups listed in the visual key. The gene groups were defined in the following way: x-linking genes *(PSO2);* NER *(RAD2, RAD4, RAD10, RAD1,* and *RAD14);* PRR *(RAD6, RAD18,* and *RAD5);* error-prone TLS *(REV1, REV3,* and *REV7);* HRR *(RAD57, RAD55, RAD51, RAD52, RAD54,* and *RAD59);* stalled replication-fork repair *(MUS81* and *MMS4).* Those compounds that form ICLs are labeled with an asterisk.

Previous studies have suggested that ICL tolerance in yeast can be attributed to three major pathways: NER, homologous recombination repair (HRR), and post-replication repair (PRR) [18,35,40]. Our results corroborate these findings as strains carrying deletions in genes of the NER *(RAD2, RAD4, RAD10, RAD1,* and *RAD14),* HRR *(RAD57, RAD55, RAD51, RAD52, RAD54,* and *RAD59),* and PRR *(RAD6, RAD18,* and *RAD5)* pathways were found to be highly sensitive to each of the six ICL-inducing compounds we tested. Figure 3 depicts the relative requirement for each of several DNA-repair modules. Although the genes that make up these modules are somewhat arbitrarily assigned, we believe that they are generally acceptable in the DNA-damage community and, moreover, provide a simple visual depiction of some of the important differences between compounds. Figure 3 provides only an overview (for more detail, see Supporting Information). Examining the top 30 sensitive strains for a given compound and the relative sensitivity rankings for these strains gives a snapshot of the first line of defense against the damage induced by that compound. The gene groups with the lowest ranks contain genes that, when deleted, result in the greatest sensitivity to that drug and therefore are presumably most important in conferring resistance. Extending this list to the top 250 sensitive strains then begins to show a more general view of other DDR pathways that are also involved in responding to damage caused by that agent.

The genes from these pathways are also important for resistance to additional types of DNA damage, as is evidenced by the profiles of other compounds (Figure 3). Therefore, their sensitivity to cross-linking agents could be due to a combined deficiency in the repair of ICLs as well as other DNA lesions. To identify strains whose sensitivity was entirely due to a lack of ICL repair, we screened the compounds angelicin and 2-DMAEC, monofunctional analogues of psoralen and mechlorethamine, respectively. The most striking difference between these profiles was the relative sensitivity of the *pso2Δ* deletion strain, which ranked among the most sensitive strains in psoralen and mechlorethamine (and indeed all other cross-linking compounds), but which exhibited no detectable sensitivity to angelicin or 2-DMAEC. These observations are consistent with previous findings for *PSO2* [41], whose protein product is thought to facilitate the repair of ICLs as a part of the NER pathway [40]. Further comparisons between ICL and non-ICL-inducing compounds underscored the importance of translesion DNA synthesis (TLS) to the repair of ICLs. This is consistent with previous findings for *REV1, REV3,* and *REV7,* which operate in a branch of PRR [42,43]. Unlike *PSO2,* however, the role of these genes in the DDR is unlikely to be restricted to ICL repair [44].

Of the six ICL-inducing compounds, carboplatin and mitomycin C resistance required a significantly larger number of genes whose biological function was not previously linked to DNA metabolism. Generally speaking, however, with respect to well-characterized DNA-damage-responsive pathways, there was a high level of correlation between the genetic requirements for resistance among the ICL-inducing compounds that we profiled. There were, however, some exceptions to this. Genes encoding the MRX complex *(MRE11, RAD50,* and *XRS2),* involved in the repair of DNA double-strand breaks [45], were particularly important for resistance to mechlorethamine (see Supporting Information). Strains deleted for the genes *MUS81* and *MMS4,* whose protein products are thought to form a structure-specific endonuclease important for restarting stalled replication forks [46,47], were highly sensitive to each cross-linker with the exception of psoralen. Finally, resistance to carboplatin appeared less dependent on NER machinery when compared to other cross-linkers, including other members of the platinum family. This is surprising because the mechanism of action and the spectrum of clinical activity of cisplatin and carboplatin are more similar to each other than to oxaliplatin [48].

### Non-Cross-Linking DNA-Damaging Agents

We also profiled five compounds that covalently modify DNA but do not induce ICLs. These included the aforementioned angelicin and 2-DMAEC, as well as streptozotocin, MMS, and 4-NQO. Streptozotocin and MMS are both clinically used cytotoxics that methylate DNA primarily at the N-7 position of guanine residues. They also generate, albeit at a lower frequency, the more toxic O-6 methylguanine and N-3 methyladenine adducts [49,50]. 4-NQO reacts with DNA to form several high-molecular-weight adducts including 3-(deoxyguanosine-N2-yl)-4-aminoquinoline-1-oxide, N-(deoxyguanosine-C8-yl)-4-aminoquinoline-1-oxide, and 3-(deoxyadenosine-N6-yl)-4-aminoquinoline-1-oxide [51].

The fitness profiles of these five DNA-modifying compounds were similar in so far as strains deficient in HRR, PRR, and *MUS81*/*MMS4* exhibited sensitivity to all five compounds (Figure 3). This likely reflects a common ability of these compounds to cause lesions that impede the advancement of DNA-replication forks during S-phase. There were also several differences in the five fitness profiles. To remove DNA adducts, the cell can employ one of several excision-repair mechanisms, the selection of which is based on the size of the offending DNA adduct [52]. In agreement with this, we found that NER was important for resistance to compounds that cause bulkier DNA adducts (angelicin and 4-NQO), whereas *MAG1,* encoding a DNA glycosylase of base excision repair [53,54], was important for resistance to the methylating activities of streptozotocin and MMS. Interestingly, *MAG1* appeared to be more important for resistance to MMS than to streptozotocin, based on rank.

A sixth non-cross-linking agent profiled, camptothecin, is distinct from the other compounds in this study because it inhibits the activity of an enzyme rather than directly modifying DNA. Camptothecin inhibits the enzyme topoisomerase I (Top I), and stabilizes a covalent enzyme–DNA intermediate [55]. During replication, collision of the replication machinery with this protein–DNA complex causes double-strand breaks. Our assay revealed a unique sensitivity profile for camptothecin. Strains deficient in the *MUS81*/*MMS4* complex and HRR showed the greatest sensitivity to camptothecin, as expected for a treatment that induces stalled replication forks. Neither PRR nor excision-repair mechanisms appeared to play a prominent role in conferring resistance to camptothecin.

### Uncharacterized Genes

Of the 36 array experiments examined in this study, 283 strains scored significantly sensitive in at least one experiment when a 5% FDR threshold was used. Among these 283 strains, 87 are sensitive in three or more independent experiments and four of these strains contain deletions in unnamed genes (Table S2). The most notable of these four strains is *YDR291W,* a gene encoding a putative DNA helicase that appears to be involved in repairing mechlorethamine-induced lesions. The amino acid sequence of Ydr291w is conserved across many organisms, including mammals (BLASTP *E*-value = 3.0e*−*08 to Human RecQ4 [56]), and its proposed helicase function is due to its helicase sequence motifs [57]; the function of this yeast open reading frame is currently being tested. Ymr073c is also conserved from yeast to man (BLASTP *E*-value = 7.0e*−*09 to a human cytochrome oxidoreductase), and our results suggest it is involved in resistance to carboplatin and cisplatin. The *ylr426wΔ* deletion strain showed sensitivity in mechlorethamine and streptozotocin, and its protein product is similar to a human peptide annotated as a dehydrogenase (BLASTP *E*-value = 6.0e*−*05). The *YKL075C* mutant strain was sensitive in both streptozotocin and camptothecin at 20 generations of exposure, and is only conserved with another fungus, *Ashbya gossypii.* All four of these unnamed genes have detectable protein expression in *S. cerevisiae* (>50 molecules/cell) [58,59].

The paucity of unnamed genes in our results is likely due to the stringency of the significance cut-off we used to minimize false positives as well as being a consequence of the level of attention that DNA-repair pathways have received. An equally important factor is the finding that many of the named genes do not properly reflect the complexity of their cellular roles. Specifically, many of the sensitive strains that carry three-letter names are poorly characterized and, more importantly, their roles in the DDR are largely unknown. For example, the *RMD7* mutant appears sensitive to several cross-linking compounds yet is named for being required for meiotic division [60]. Other genes are better characterized, but thus far are only known to have functions not involved in the DDR (e.g., *RTT101* [regulates Ty1 transposition, though other RTT genes are DDR genes, such as *ELG1, MMS1,* and *RTT107*], and *LTE1* [essential for growth in low temperatures]) [61,62]. Overall, the 283 strains making the sensitivity cut-off in at least one experiment contain 34 strains deleted for genes with no functional annotation (by Gene Ontology [63]), and roughly half are annotated in processes unrelated to DNA repair (Table S2). Our results provide functional data that these genes are in fact involved in the DDR and suggest additional experiments to further characterize them.

### Global Analysis

**Figure 4:**
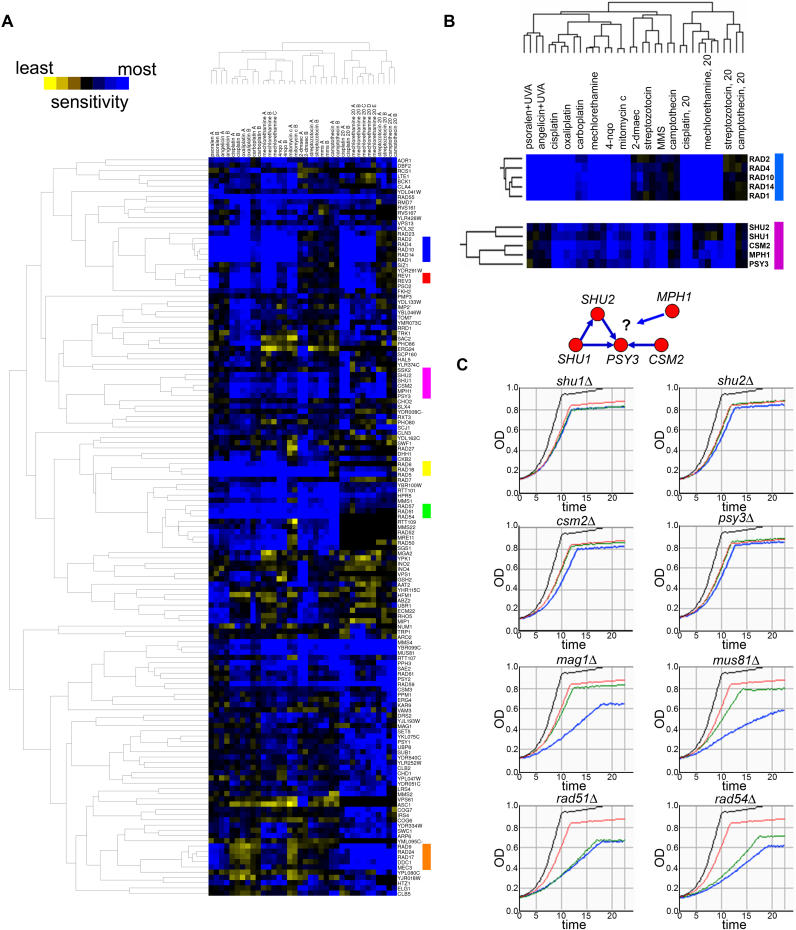
Hierarchical Clustering of Genome-Wide Profiles Identifies Mechanistic Relationships Between Drugs and Functional Relationships Between Genes (A) Clustergram containing all strains significant in two or more array experiments. Raw fitness-defect values were hierarchically clustered using Spearman's rank correlation. Colored bars represent gene clusters of note, including NER (*RAD2, RAD4, RAD10, RAD14,* and *RAD1—*blue); error-prone TLS (*REV1* and *REV3—*red); PRR (*RAD6, RAD18,* and *RAD5—*yellow); homologous recombination (*RAD57, RAD51,* and *RAD54—*green); cell-cycle checkpoint control (*RAD9, RAD24, RAD17, DDC1,* and *MEC3—*orange); and a cluster shown in (B) (*SHU2, SHU1, CSM2, MPH1,* and *PSY3—*magenta). (B) Zoom view showing one cluster containing the class I NER genes and a second cluster containing several uncharacterized DNA-repair genes. Four of these five genes *(SHU1, SHU2, CSM2,* and *PSY3)* are known to encode proteins that physically interact [65,77,78]. (C) Individual growth curves of single and double deletion strains with *MPH1* in 0.002% MMS. In all panels, the growth of wild-type (BY4741) is represented by the black curve and the growth of *mph1Δ* by the red curve. The growth of eight different deletion strains *(shu1Δ, shu2Δ, csm2Δ, psy3Δ, mag1Δ, mus81Δ, rad51Δ,* and *rad54Δ)* are shown in green, and double mutants, in which the *MPH1* deletion is added to each of the above, are shown in blue. Double mutants of *MPH1, MAG1,* and *MUS81* show additive or synergistic sensitivity to MMS, whereas double mutants of *MPH1,* with the four other genes in its cluster, show no additional sensitivity to MMS, suggesting that *MPH1* is epistatic with *SHU1, SHU2, CSM2,* and *PSY3*.

To analyze the 36 array experiments presented here in a global manner, we applied hierarchical clustering techniques to the fitness-defect scores (Figure 4A). The dataset used for clustering was restricted to strains designated as significant (5% FDR) in two or more of the array experiments, and thus represented only the most highly sensitive strains identified in this study. This produced a matrix of fitness-defect scores (141 strains × 36 experiments). An examination of the clusters across the experiment axis showed that, as expected, compounds predicted to have similar mechanisms of action group together, lending confidence in the robustness of the analysis. Members of the platinum family of compounds are highly correlated, as are MMS and streptozotocin, the only two agents profiled that possess DNA-methylating activity. The genome-wide data was highly reproducible, evidenced by the fact that all replicates correlate as nearest neighbors.

When the clustered results are viewed along the gene axis, we predicted there would be a high correlation in fitness profiles between strains with deficiencies in genes in the same DNA-repair pathway (Figure 4B), and this prediction was confirmed. This result holds true for several DNA-repair epistasis groups, including NER, PRR, HRR, error-prone TLS, and cell-cycle checkpoint control (see Figure 4A).

The clustering analysis suggested additional novel functional interactions. We focused on one particular cluster within the dataset that included five genes: *MPH1, SHU1, SHU2, CSM2,* and *PSY3*. The latter four genes encode proteins that physically interact with one another and belong to a single epistasis group that appears to operate in a branch of HRR [64,65]. *MPH1* was also shown to be epistatic with members of the HRR pathway [66], and the Mph1 protein, as predicted by its amino acid sequence, was recently shown to possess DNA helicase activity [67]. To our knowledge, however, no direct functional link between *MPH1* and the other four genes has been reported. To test whether such a link exists, as suggested by our clustering analysis, we generated double mutants between *MPH1* and each of the other four genes and measured the sensitivity of these strains to MMS. We found that single and double deletion strains exhibited similar sensitivity to MMS, indicating that *MPH1* is epistatic with *SHU1, SHU2, CSM2,* and *PSY3* (Figure 4C). Conversely, the addition of an *MPH1* deletion to strains carrying deletions known to compromise other defined DNA-damage pathways, such as *MUS81* (stalled replication-fork repair) and *MAG1* (base excision repair), exacerbated the sensitivity to MMS (Figure 4C).

## Discussion

The high level of scrutiny paid to the DDR in yeast and other model organisms has yielded a wealth of data. In this study, we set out to address two aims. First, we sought to collect a sufficient number of fitness-profile signatures for compounds with similar mechanisms of action. This would allow us to test whether we could identify differences in compound profiles that might provide correlative insight into their diverse clinical efficacy. Second, we aimed to develop a standard assay platform, including all protocols and strains so that they can be exported to any laboratory. We believe that such a standard set of protocols will provide a powerful resource to the entire community and will allow for the development of a centralized database for the exchange of information.

While classical forward genetic screens in yeast have been instrumental in identifying important components of the DDR, it is unlikely that saturation across the genome has been achieved. In contrast to traditional genetic analysis, the approach employed here enabled the systematic interrogation of the comprehensive set of all 4,758 homozygous deletion strains, representing nearly all non-essential genes in the yeast genome. Furthermore, because the fitness-defect scores obtained with this method display a continuous range of sensitivity and these sensitivities correlate well with independently measured growth rates (see Figure 1B), genes can be ranked according to their relative importance in conferring resistance to a given compound. This provides a high level of resolution and allows distinctions to be made between functionally related compounds, even in cases where the genetic elements required for fitness largely overlap. For example, cisplatin, oxaliplatin, and carboplatin are structurally related chemotherapeutic drugs and, for each, their cytotoxicity is attributed primarily to their ability to induce ICLs. Predictably, fitness profiling of each compound identified the major pathways important for resistance to DNA cross-linking agents (*PSO2*/NER, HRR, and PRR). The fitness profile for carboplatin, however, was unique among the platinum-based compounds in that PRR was found to hold far greater importance in conferring resistance than the other pathways (see Figure 3). Carboplatin's distinctive profile is also apparent upon hierarchical clustering of the data (see Figure 4A), and could potentially reflect differences in the spectrum of structural damage to DNA or in cell-cycle-specific reactivity of the drug.

Although DNA-targeting drugs are typically viewed as non-specific, the cellular response to both diverse and functionally similar DNA-damaging agents is clearly distinct as is evidenced by their specific clinical applications [68]. It is important to note that most of these agents were used before their mechanism of action had been defined, and their effectiveness is still defined empirically. Given this fact and the fact that DNA will remain an excellent therapeutic oncology target, particularly as more selective and less toxic compounds are developed, high-throughput assays capable of defining drug action will undoubtedly prove useful. In this study, we used 12 compound profiles (Table 1) to define a benchmark from which the application of this assay to novel DNA-damaging compounds can be compared.

Because the measured sensitivity of each strain in a given profile is a continuum of sensitivity as measured by growth inhibition, it is difficult to gauge the point at which fitness-defect scores cease to reflect significant defects in growth. We therefore sought to confirm the sensitivity detected in competitive pools by growing individual strains +/− the compound mechlorethamine. We found that 88% of the 233 strains most sensitive to mechlorethamine (based on rank) were also found to be sensitive in our individual growth assay (see Table 2). Thus, our assay has the advantage of having a measurable FDR that appears much lower than our calculated FDR [69]. This result is significant on several levels. First, we found 34 uncharacterized genes suggesting, as predicted, that our understanding of the DDR is still incomplete. Second, these results suggest that by choosing a stringent significance cut-off, we have likely missed other DDR genes, and the raw data from these screens should provide a rich data source for further analysis. Added to this complexity is our observation that several strains exhibit compound sensitivity only following extended drug exposure. By conducting our assay for both five and 20 generations of growth, we have gleaned a more comprehensive representation of the DDR. This enabled us to establish definitively a role for five checkpoint genes in conferring resistance to cisplatin (see Figure 2)—an observation for which there has been no consensus in the literature.

Our assay is distinct from expression studies, where an equivalent confirmation assay would represent hundreds of Northern blots or quantitative PCR assays to verify expression changes. In addition, expression arrays do not allow a ranking of genes as it is not clear how or if the magnitude of expression change is biologically significant. The fact that our assay allows a ranking of sensitivities, and confirmation of this ranking, makes it distinct from several colony-based studies using the yeast deletion collection. These solid-media assays for sensitivity are not highly quantitative, though spot-dilution confirmation does allow some measure of relative sensitivities.

In summary, despite significant advances in the breadth and depth of our understanding of the DDR, additional cell-based analyses are required before we can claim to fully understand DNA replication, recombination, and repair [70]. We believe that a new theme has emerged in these studies, however, in that the combined use of chemicals with genetic deletions can define functional groups that, in the absence of compound, would not be uncovered. For example, hierarchical clustering of the most highly sensitive strains identified using a combination of chemical and genetic perturbations successfully predicted novel epistatic interactions between the *MPH1* gene and *SHU1, SHU2, CSM2,* and *PSY3*. Even though they comprise a major defense mechanism against several DNA-damaging agents, a role for these five gene products in a branch of HRR is only now beginning to emerge [65,66]. Further analysis of this dataset will undoubtedly uncover additional functional relationships whose discovery has thus far remained elusive. Finally, increased use (and increased availability) of chemical inhibitors to probe the yeast deletion collection should provide a more comprehensive, contextual understanding of cellular physiology.

## Materials and Methods

### Reagents.

Cisplatin, carboplatin, oxaliplatin, psoralen, angelicin, streptozotocin, 2-DMAEC, mechlorethamine, MMS, camptothecin, and 4-NQO were purchased from Sigma-Aldrich (St. Louis, Missouri, United States). Mitomycin C was purchased from Calbiochem (San Diego, California, United States). Each of these compounds was dissolved in DMSO, aliquoted, and stored at −20 °C until use, with the exception of cisplatin, which was used immediately following resuspension, and carboplatin, oxaliplatin, and 2-DMAEC, which were dissolved directly in YPD media at the time of the experiment.

### Strains and media.

Yeast was maintained in YPD media [71,72] at 30 °C unless stated otherwise. Strains used for individual analysis in this study are listed in Table S3 and were obtained from the yeast deletion collection or constructed de novo using PCR-based gene replacement [73]. Using this method, the *nat* or *hph* genes, conferring resistance to the antibiotics nourseothricin or hygromycin B, respectively [74], were PCR-amplified from plasmids and transformed into the appropriate background strain using a modified LiOAc-based protocol [75,76]. Transformants were selected on YPD containing antibiotic (100 μg/ml nourseothricin or 300 μg/ml hygromycin B), and successful gene-replacement events were verified by PCR.

### Deletion-pool construction and growth.

The homozygous deletion pool was constructed as described [22] and stored in 20-μl aliquots at −80 °C. For the five-generation experiments, aliquots of the pool were thawed and diluted in YPD to an optical density at 600 nm (OD_600_) of 0.0625 and a final volume of 700 μl. Compound was then added and the pool was grown for five generations in a Tecan GENios microplate reader (Tecan, Durham, North Carolina, United States). For the 20-generation experiments, the thawed pool was first recovered for ten generations of logarithmic growth, diluted as above, and grown in the presence of compound over 20 generations. Cells were maintained in logarithmic phase by robotically diluting cultures every five doublings using a Packard Multiprobe II four-probe liquid-handling system (PerkinElmer, Wellesley, California, United States) [23].

Experiments involving the two UVA light-activated compounds (psoralen and angelicin) were performed as described above with the following modifications: the pool was diluted to OD_600_ of 0.625 and compound was added to 70 μl of this cell suspension, which was then exposed to UVA light via a handheld UVA lamp (Ambion, Austin, Texas, United States) for 15 min. Following irradiation, cells were diluted 10× with YPD to a final volume of 700 μl and grown as described above.

### Genomic DNA preparation, PCR, and microarray hybridization.

Genomic DNA preparation, PCR amplification of molecular tags, and microarray hybridization were as described (Datasets S3 and S4; Protocol S3) [23].

### Data analysis.

Fitness-defect scores were calculated for each strain in the pool for each experiment. These scores are based on a tag-specific algorithm that takes into account the intensities of each tag on the experimental array and the corresponding intensities on a set of control arrays performed on the pool without compound (the control set). The majority of strains carry four tags that hybridize to the array: upstream tag (uptag), uptag antisense, downstream tag (dntag), and dntag antisense. The tag intensities are log transformed, mean normalized, and the intensities of the two strands of each tag are averaged into a single value for each tag. Next, a mean and standard deviation are calculated for the uptag and dntag intensities for each strain across the set of control arrays. A *z*-score for upstream and downstream tags for each strain is then calculated by taking the difference of the average intensities between the control and treatment and dividing by the standard deviation of the control-set intensities. The result is two *z*-score values for the upstream and downstream tags; these are then averaged into a single fitness-defect score for the strain. A significance cut-off for each experiment was determined by calculating the score cut-off required for a 0.05 FDR [28,29].

### Clustering analysis.

Hierarchical clustering analysis was performed with Cluster 3.0 (http://bonsai.ims.u-tokyo.ac.jp/~mdehoon/software/cluster/software.htm) in Windows and Linux and visualized using Java Treeview (http://jtreeview.sourceforge.net/) and slcview (http://slcview.stanford.edu). Clustering along both experiment and gene axes was performed on the calculated fitness-defect scores (as described above) using average linkage and Spearman's rank correlation as a distance metric (Dataset S5).

### Confirmations of individual strain growth rates.

Individual yeast strains were first grown to saturation for approximately 20 h. Cells were then diluted to an OD_600_ of 0.02 in a final volume of 100 μl using a Biomek FX Laboratory Automation Workstation (Beckman Coulter, Allendale, New Jersey, United States). Normalized cultures were grown in 96-well plates (Nunc, Rochester, New York, United States) in Tecan GENios microplate readers (Tecan) for up to 30 h. The growth rate of each culture was monitored by measuring the OD_600_ every 15 min and calculating the average doubling time (AvgG). AvgG was calculated by recording the time (Δ*t*) from the start of growth until the optical density of the culture reached the calibrated five-generation point (OD5g) and dividing this by the number of generations, i.e., five. If the culture did not reach OD5g, AvgG was calculated by using a binomial search to determine the fractional generations of the optical density at the end of the growth (ODf), assuming an exponential growth rate, then dividing the time to ODf by the generations calculated.

We defined the sensitivity to mechlorethamine of each individual strain as the difference between the AvgG in mechlorethamine and in DMSO (ΔAvgG = AvgG_mech_ − AvgG_DMSO_). Individual deletion strains were scored as sensitive to mechlorethamine if this difference was greater than that of wild-type +/− the standard deviation calculated from 18 wild-type replicates.

### Viability assays.

Selected strains were grown for five generations in the presence of compound and cells collected. Cells were transferred to 96-well plates and 5-fold dilutions were prepared. These dilutions were then “stamped” onto YPD plates using a pintool calibrated to deliver 5 μl. Cells were then allowed to form colonies for 2 d at 30 °C.

## Supporting Information

The supporting files are also available in a searchable format at http://chemogenomics.stanford.edu.

Dataset S1 Analyzed Microarray Data Calculated fitness-defect scores for each microarray experiment are presented.
(7.2 KB XLS)

Dataset S2 Complete Set of Individual Strain Growth-Curve Data Each strain has three growth curves corresponding to growth in 1% DMSO, 31.3 μM mechlorethamine, and 62.5 μM mechlorethamine, respectively.
(1.7 MB PNG)

Dataset S3 Unanalyzed, Raw Microarray Data from Affymetrix Software, Part 1 This file contains 18 .cel files output from Affymetrix GeneChip operating system; these files constitute half of the total microarray dataset.
(9.2 MB ZIP)

Dataset S4 Unanalyzed, Raw Microarray Data from Affymetrix Software, Part 2 This file contains 18 .cel files output from Affymetrix GeneChip Operating System; these files constitute half of the total microarray dataset.
(8.5 MB ZIP)

Dataset S5 Files Containing Cluster Output Used in Generating Figure 4A These text files can be opened by clustergram-viewing software to browse the cluster shown in Figure 4A.
(24 KB TXT)

Protocol S1 Document Describing the Data Included in Dataset S1
(26 KB DOC)

Protocol S2 Document Describing the Data Included in Dataset S2
(24 KB DOC)

Protocol S3 Document Describing the Data Included in Datasets S3 and S4
(84 KB DOC)

Table S1 Strains Significantly Sensitive in Two or More 20-Generation Experiments that Exhibit Greater Sensitivity in Long-Term Drug Exposure (61 KB DOC)

Table S2 Number of Times Strains Were Calculated as Significantly Sensitive for Each Drug Condition and Length of Drug Exposure (80 KB XLS)

Table S3 Individual Strains Used in this Study (39 KB DOC)

### Accession Numbers

Swiss-Prot (http://www.ebi.ac.uk/swissprot) accession numbers for the strains discussed in this paper are as follows: *CSM2* (P40465), *DDC1* (Q08949), *ELG1* (Q12050), *LTE1* (P07866), *MAG1* (P22134), *MEC3* (Q02574), *MMS1* (Q06211), *MMS4* (P38257), *MPH1* (P40562), *MRE11* (P32829), *MUS81* (Q04149), *PSO2* (P30620), *PSY3* (Q12318), *RAD1* (P06777), *RAD2* (P07276), *RAD4* (P14736), *RAD5* (P32849), *RAD6* (P06104), *RAD9* (P14737), *RAD10* (P06838), *RAD14* (P28519), *RAD17* (P48581), *RAD18* (P10862), *RAD24* (P32641), *RAD50* (P12753), *RAD51* (P25454), *RAD52* (P06778), *RAD54* (P32863), *RAD55* (P38953), *RAD57* (P25301), *RAD59* (Q12223), *REV1* (P12689), *REV3* (P14284), *REV7* (P38927), *RMD7* (P40056), *RTT101* (P47050), *RTT107* (P38850), *SHU1* (P38751), *SHU2* (P38957), *YDR291W* (Q05549), *YKL075C* (P36083), *YLR426W* (Q06417), *YMR073C* (Q04772), and *XRS2* (P33301).

## Abbreviations

DDR: DNA-damage response
2-DMAEC: 2-dimethylaminoethyl chloride
DMSO: dimethyl sulfoxide
FDR: false-discovery rate
HRR: homologous recombination repair
ICL: interstrand cross-link
MMS: methyl methanesulfonate
NER: nucleotide excision repair
4-NQO: 4-nitroquinoline-1-oxide
PRR: post-replication repair
TLS: translesion DNA synthesis
YPD: yeast extract/peptone/dextrose
