# Supplementary material for: Genome-Wide Requirements for Resistance to Functionally Distinct DNA-Damaging Agents
Source: PLoS Genet. 2005 Aug 19;1(2):e24. doi: 10.1371/journal.pgen.0010024 (PMC1189734; doi:10.1371/journal.pgen.0010024)
Supplement: Supplementary file 3 [file pgen.0010024.sd006.doc]

**Dataset S1**. Analyzed ORF/Gene Microarray Data

The data herein represents the final calculated fitness defect values for each homozygous diploid deletion strain rich media (ypd) in the presence of drug. All fitness defect values listed here were generated from the individual tag intensity values as described in the manuscript. Values of 0 were not calculated.

The headings indicate:

- **ORF** - the systematic *S. cerevisiae* open reading frame designation
- **Batch** - Yeast Deletion Project designation for strain batch
- **Gene** - the gene name, if available
- **Go_process, Go_function, Go_component** - Gene Ontology descriptions
- **deletion_phenotype** - viability phenotype of the deletion strain
- **result_np** - the fitness defect score for the designated experiment, higher scores mean greater sensitivity
- **significant_np** - 1 means this strain's fitness defect score exceeded the 0.05 false discovery rate threshold
- **rank_np** - rank of the fitness defect score for this strain among the others in this experiment, rank 1 is the most sensitive strain
- **result_np, significant_np**, and **rank_np** all refer to individual experiments in the format of filename:drug:concentration:units::::pool:generations

  e.g. 04_02_24_05:cisplatin:500:uM::::hom_09_02:5
  designates the data for file 04_02_24_05.cel, which was the pool grown in cisplatin at 500 uM concentration for 5 generations of growth.

* See the manuscript for details on how the result_np fitness defect score and the significant_np significance call were calculated.
