# Supplementary material for: Genome-Wide Requirements for Resistance to Functionally Distinct DNA-Damaging Agents
Source: PLoS Genet. 2005 Aug 19;1(2):e24. doi: 10.1371/journal.pgen.0010024 (PMC1189734; doi:10.1371/journal.pgen.0010024)
Supplement: Supplementary file 4 [file pgen.0010024.sd007.doc]

**Dataset S2**. Individual Strain Growth Curve Data

Three 96-well plates containing individual yeast deletion strains were grown in an automated incubator/spectrophotometer and OD600 readings were taken every 15 minutes for 95 reads.

Each plate was grown in DMSO (diluent control), 31.3uM of mechlorethamine, and 62.5uM of mechlorethamine for a total of 9 96-well plates worth of growth.

The identity of the strain in each well of each plate is noted in mech_hd_plate_layouts.txt, the three plates are named mech_hd1, mech_hd2, and mech_hd3 representing that the strains were picked for likely sensitivity to mechlorethamine as based on the array data, and they are all homozygous diploid deletion strains.

Each image contains all 96 curves (plus reference curves) for that plate. In each graph, the red curve represents that strain grown in DMSO as a reference, and the black curve is the growth of the strain in drug.
