# Supplementary material for: Genome-Wide Requirements for Resistance to Functionally Distinct DNA-Damaging Agents
Source: PLoS Genet. 2005 Aug 19;1(2):e24. doi: 10.1371/journal.pgen.0010024 (PMC1189734; doi:10.1371/journal.pgen.0010024)
Supplement: Supplementary file 5 [file pgen.0010024.sd008.doc]

**Dataset S3**. Raw Microarray Data

All raw data is available as Affymetrix .cel files, listing coordinates and intensity values. The filenames correspond to experiments as follows.

| filename | drug | concentration | generations | short_name (Fig 4A) |
| --- | --- | --- | --- | --- |
| 04_02_24_05 | cisplatin | 500uM | 5 | cisplatin A |
| 04_03_17_06 | cisplatin | 500uM | 5 | cisplatin B |
| 03_04_04_03 | cisplatin | 500uM | 20 | cisplatin 20 A |
| 03_04_04_04 | cisplatin | 500uM | 20 | cisplatin 20 B |
| 04_03_09_01 | carboplatin | 15mM | 5 | carboplatin A |
| 04_03_30_02 | carboplatin | 15mM | 5 | carboplatin B |
| 04_02_24_07 | oxaliplatin | 4mM | 5 | oxaliplatin A |
| 04_03_17_07 | oxaliplatin | 4mM | 5 | oxaliplatin B |
| 04_03_17_10 | psoralen irradiated | 0.5uM | 5 | psoralen A |
| 04_03_25_05 | psoralen irradiated | 0.5uM | 5 | psoralen B |
| 04_03_17_12 | angelicin irradiated | 62.5uM | 5 | angelicin A |
| 04_03_25_06 | angelicin irradiated | 62.5uM | 5 | angelicin B |
| 03_12_09_16 | mechlorethamine | 62.5uM | 5 | mechlorethamine A |
| 03_12_19_01 | mechlorethamine | 62.5uM | 5 | mechlorethamine B |
| 04_01_21_09 | mechlorethamine | 62.5uM | 5 | mechlorethamine C |
| 02_12_18_13 | mechlorethamine | 62.5uM | 20 | mechlorethamine 20 A |
| 03_02_05_04 | mechlorethamine | 62.5uM | 20 | mechlorethamine 20 B |
| 03_02_05_05 | mechlorethamine | 62.5uM | 20 | mechlorethamine 20 C |
| 03_03_20_07 | mechlorethamine | 62.5uM | 20 | mechlorethamine 20 D |
| 03_03_20_08 | mechlorethamine | 62.5uM | 20 | mechlorethamine 20 E |
| 04_03_09_06 | 2-dmaec | 240mM | 5 | 2-dmaec A |
| 04_03_17_15 | 2-dmaec | 240mM | 5 | 2-dmaec B |
| 04_02_24_02 | mitomycin c | 1mM | 5 | mitomycin c A |
| 04_03_30_03 | mitomycin c | 1mM | 5 | mitomycin c B |
| 04_03_17_16 | streptozotocin | 2mM | 5 | streptozotocin A |
| 04_03_25_07 | streptozotocin | 2mM | 5 | streptozotocin B |
| 03_10_08_13 | streptozotocin | 2mM | 20 | streptozotocin 20 A |
| 04_07_16_09 | streptozotocin | 2mM | 20 | streptozotocin 20 B |
| 04_02_24_03 | mms | 0.02% | 5 | mms A |
| 04_03_25_04 | mms | 0.02% | 5 | mms B |
| 04_03_25_01 | camptothecin | 30ug/mL | 5 | camptothecin A |
| 04_03_30_01 | camptothecin | 30ug/mL | 5 | camptothecin B |
| 04_05_12_10 | camptothecin | 30ug/mL | 20 | camptothecin 20 A |
| 04_07_16_08 | camptothecin | 30ug/mL | 20 | camptothecin 20 B |
| 04_08_03_07 | 4nqo | 0.0313uM | 5 | 4nqo A |
| 04_08_05_01 | 4nqo | 0.0313uM | 5 | 4nqo B |

The information needed to translate cel file information into orf_tag information can be found in the Chip_Info_table.txt file. Only the coordinates used in the data analysis are listed. The headers are:

- **x** - x-coordinate
- **y** - y-coordinate
- **oligo** - oligonucleotide sequence, 3' to 5' direction
- **orf_name** - orf name
- **up_dntag** - lists whether the gene is uptag or dntag
- **sense** - denotes whether the tag on the chip is in the sense direction (meaning it hybridizes to the strand amplified from the kan cassette B-U2 comp or kan cassette B-D2 comp) or antisense direction (meaning it hybridizes to the strand amplified from B-U1 or B-D1). B-U1, B-U2 comp, B-D1, B-D2 comp are defined in Giaever et al. Nature 418, 387-391 (2002).
