# Supplementary material for: Genome-Wide Requirements for Resistance to Functionally Distinct DNA-Damaging Agents
Source: PLoS Genet. 2005 Aug 19;1(2):e24. doi: 10.1371/journal.pgen.0010024 (PMC1189734; doi:10.1371/journal.pgen.0010024)
Supplement: Supplementary file 6 [file pgen.0010024.st001.doc]

**Table S1**. Strains significantly sensitive in two or more 20 generation experiments that exhibit greater sensitivity in long-term drug exposure.

| **ORF** | **GENE** | **# 5 sig** | **# 20 sig** |
| --- | --- | --- | --- |
| YLR288C | MEC3 | 0 | 10 |
| YER173W | RAD24 | 1 | 9 |
| YOR368W | RAD17 | 2 | 9 |
| YDR217C | RAD9 | 2 | 7 |
| YKL076C | YKL076C | 0 | 6 |
| YNL201C | PSY2 | 1 | 6 |
| YPL194W | DDC1 | 2 | 6 |
| YGL087C | MMS2 | 0 | 5 |
| YIL153W | RRD1 | 0 | 5 |
| YMR214W | SCJ1 | 0 | 5 |
| YGL005C | COG7 | 0 | 4 |
| YPR119W | CLB2 | 0 | 4 |
| YAL011W | SWC3 | 0 | 3 |
| YKL075C | YKL075C | 0 | 3 |
| YKR019C | IRS4 | 0 | 3 |
| YLR085C | ARP6 | 0 | 3 |
| YMR048W | CSM3 | 0 | 3 |
| YNL070W | TOM7 | 1 | 3 |
| YHL006C | SHU1 | 2 | 3 |
| YBL046W | YBL046W | 0 | 2 |
| YDL076C | RXT3 | 0 | 2 |
| YDR007W | TRP1 | 0 | 2 |
| YDR435C | PPM1 | 0 | 2 |
| YDR439W | LRS4 | 0 | 2 |
| YDR484W | VPS52 | 0 | 2 |
| YER164W | CHD1 | 0 | 2 |
| YGL012W | ERG4 | 0 | 2 |
| YHR207C | SET5 | 0 | 2 |
| YJL165C | HAL5 | 0 | 2 |
| YJL193W | YJL193W | 0 | 2 |
| YLR135W | SLX4 | 0 | 2 |
| YLR252W | YLR252W | 0 | 2 |
| YMR039C | SUB1 | 0 | 2 |
| YMR223W | UBP8 | 0 | 2 |
| YNL068C | FKH2 | 0 | 2 |
| YNR031C | SSK2 | 0 | 2 |
| YOL001W | PHO80 | 0 | 2 |
| YOR106W | VAM3 | 0 | 2 |
| YPL047W | SGF11 | 0 | 2 |
| YPL080C | YPL080C | 0 | 2 |
| YPL269W | KAR9 | 0 | 2 |
| YDR014W | RAD61 | 1 | 2 |
| YGL148W | ARO2 | 1 | 2 |
| YGR092W | DBF2 | 1 | 2 |
| YMR073C | YMR073C | 1 | 2 |
| YNL041C | COG6 | 1 | 2 |
