# Supplementary material for: Genome-Wide Requirements for Resistance to Functionally Distinct DNA-Damaging Agents
Source: PLoS Genet. 2005 Aug 19;1(2):e24. doi: 10.1371/journal.pgen.0010024 (PMC1189734; doi:10.1371/journal.pgen.0010024)
Supplement: Supplementary file 7 [file pgen.0010024.st003.doc]

| **Strain** | **Genotype** | **Source** |
| --- | --- | --- |
| BY4741 | MATa his3Δ1 leu2Δ0 met15Δ0 ura3Δ0 | Giaever et al. 2002* |
| HHY300 | BY4741; mph1Δ::Hyg |  |
|  | BY4741; mus81Δ::Kan | Giaever et al. 2002* |
| HHY301 | BY4741; mus81Δ::Kan mph1Δ::Hyg |  |
|  | BY4741; mag1Δ::Kan | Giaever et al. 2002* |
| HHY302 | BY4741; mag1Δ::Kan mph1Δ::Hyg |  |
|  | BY4741; rad51Δ::Kan | Giaever et al. 2002* |
| HHY303 | BY4741; rad51Δ::Kan mph1Δ::Hyg |  |
|  | BY4741; rad54Δ::Kan | Giaever et al. 2002* |
| HHY304 | BY4741; rad54Δ::Kan mph1Δ::Hyg |  |
|  | BY4741; shu1Δ::Kan | Giaever et al. 2002* |
| HHY309 | BY4741; shu1Δ::Kan mph1Δ::Hyg |  |
| HHY305 | BY4741; shu2Δ::Nat |  |
| HHY306 | BY4741; shu2Δ::Nat mph1Δ::Hyg |  |
|  | BY4741; psy3Δ::Kan | Giaever et al. 2002* |
| HHY307 | BY4741; psy3Δ::Kan mph1Δ::Hyg |  |
|  | BY4741; csm2Δ::Kan | Giaever et al. 2002* |
| HHY308 | BY4741; csm2Δ::Kan mph1Δ::Hyg |  |

**Table S3**. Individual strains used in this study

* Strains referenced to Giaever et al. 2002 are from the Yeast Deletion Collection and are referring to:

Giaever G, Chu AM, et al. (2002) Functional Profiling of the Saccharomyces cerevisiae genome. Nature Jul 25;418(6896):387-91.

All other strains were constructed for this study.
